# Supplementary material for: Development of Carbazole Derivatives Compounds against Candida albicans: Candidates to Prevent Hyphal Formation via the Ras1-MAPK Pathway
Source: J Fungi (Basel). 2021 Aug 25;7(9):688. doi: 10.3390/jof7090688 (PMC8466151; doi:10.3390/jof7090688)
Supplement: Supplementary file 1 [file jof-07-00688-s001.zip › jof-1310716-supplementary/Figures S1-S4.pdf]

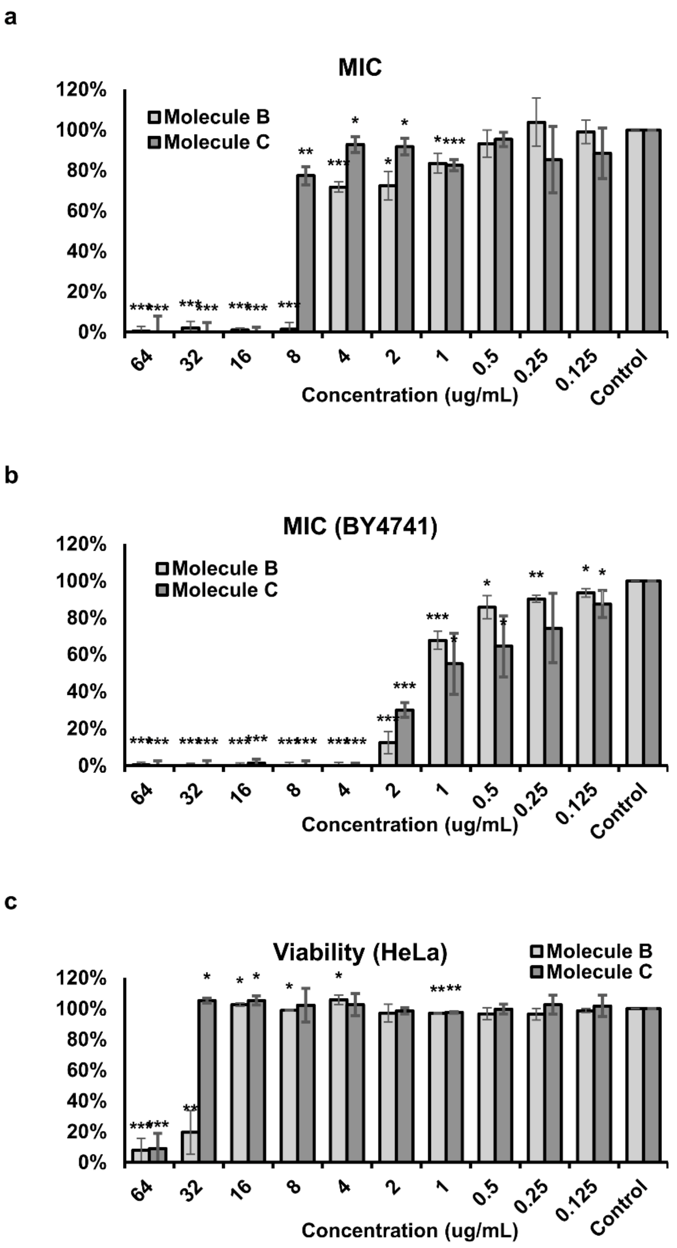

Figure S1. The cytotoxicity of Molecule B and Molecule C in *C. albicans*, *S. cerevisiae*, and mammalian cell. (**a**) The cytotoxicity of Molecule B and Molecule C on *C. albicans*. SC5314 strain cells (2×10^4 cells/mL) were grown with Molecule B and Molecule C in RPMI1640 medium with 0.165M MOPS for 24 h at 37°C according to the CLSI guidelines [14]. The growth inhibition was determined by measuring the optical density at 595 nm using a microplate reader. (**b**) The cytotoxicity of Molecule B and Molecule C on *S. cerevisiae*. BY4741 strain cells (2×10^4 cells/mL) were grown with Molecule B and Molecule C in YPD medium 24 h at 30°C. The growth inhibition was determined by measuring the optical density at 595 nm using a microplate reader. (**c**) The cytotoxicity of Molecule B and Molecule C on mammalian cells. Viability was measured based on the MTS assay. Each well was inoculated with HeLa cells (10^5 cells/mL). After incubation at 37°C for 24 h in DMEM medium containing 10% fetal bovine serum. The final concentrations of Molecule B and Molecule C (ranging from 0.125–64 μg/mL) were added and incubated at 37°C for 16 h. The absorbance at 490 nm was measured with a microtiter plate reader. Each experiment was conducted in triplicate. The data represent the mean and standard deviation of three independent experiments. *; $P<0.05$, **; $P<0.01$, ***; $P<0.001$ (*t*-test).

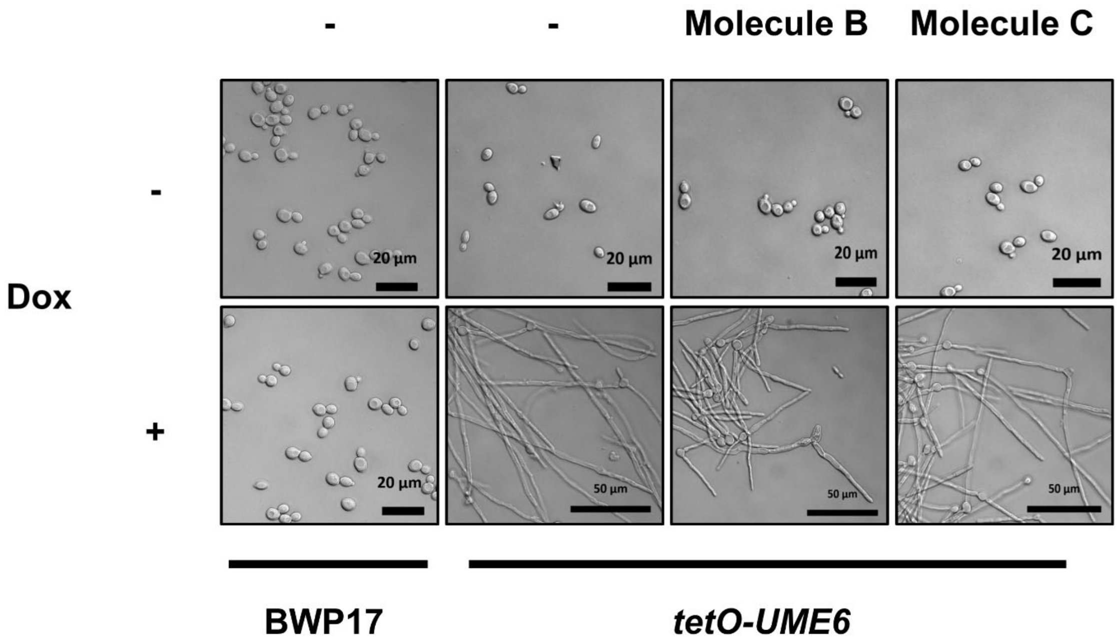

Figure S2. Morphology of the tetO-*UME6* strain treated with Molecule B and Molecule C with and without doxycycline. For overnight cultivation at 30°C in YPD, the cells were inoculated with or without dox in a fresh YPD medium containing or without Molecule B and Molecule C and incubated with shaking at 30°C for 2 h. The morphology of the cells was photographed with a microscope. Scale bars represent 10μm(yeast form) and 20μm(hyphae).

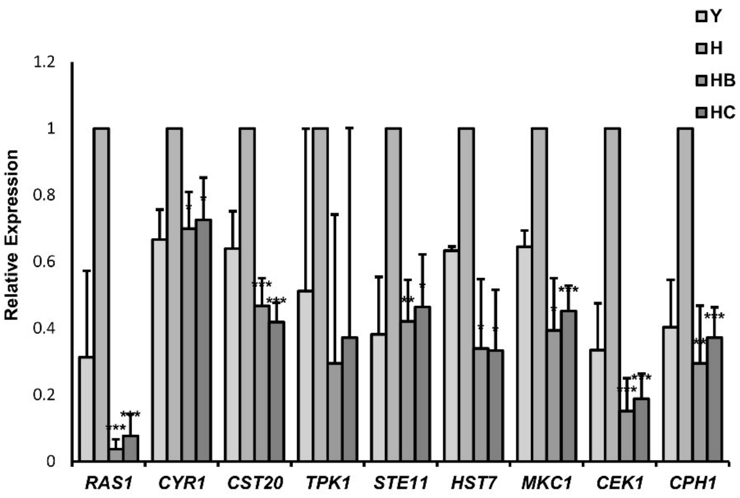

Figure S3. Quantitative RT-PCR analysis of MAPK cascade-related genes in *C. albicans*.

Yeast cells were cultivated at 30°C in a YPD medium and morphogenesis-induced cells were incubated with or without treatment with Molecule B and Molecule C at 37°C in a YPD medium containing 10% FBS. Total RNA of yeast cells (Y), Molecule B and Molecule C treated hyphae-induced cells (HB and HC, respectively), or hyphae-induced cells (H) were isolated using the TRIzol reagent method. The expression of RAS1 and MAPK cascade-related genes was investigated using qRT-PCR and the primers listed in Supplemnetray table 1. Data information: Each experiment was conducted in triplicate. The data represent the mean and standard deviation of three independent experiments. *; P<0.05, **; P<0.01, ***; P<0.001 (*t*-test).

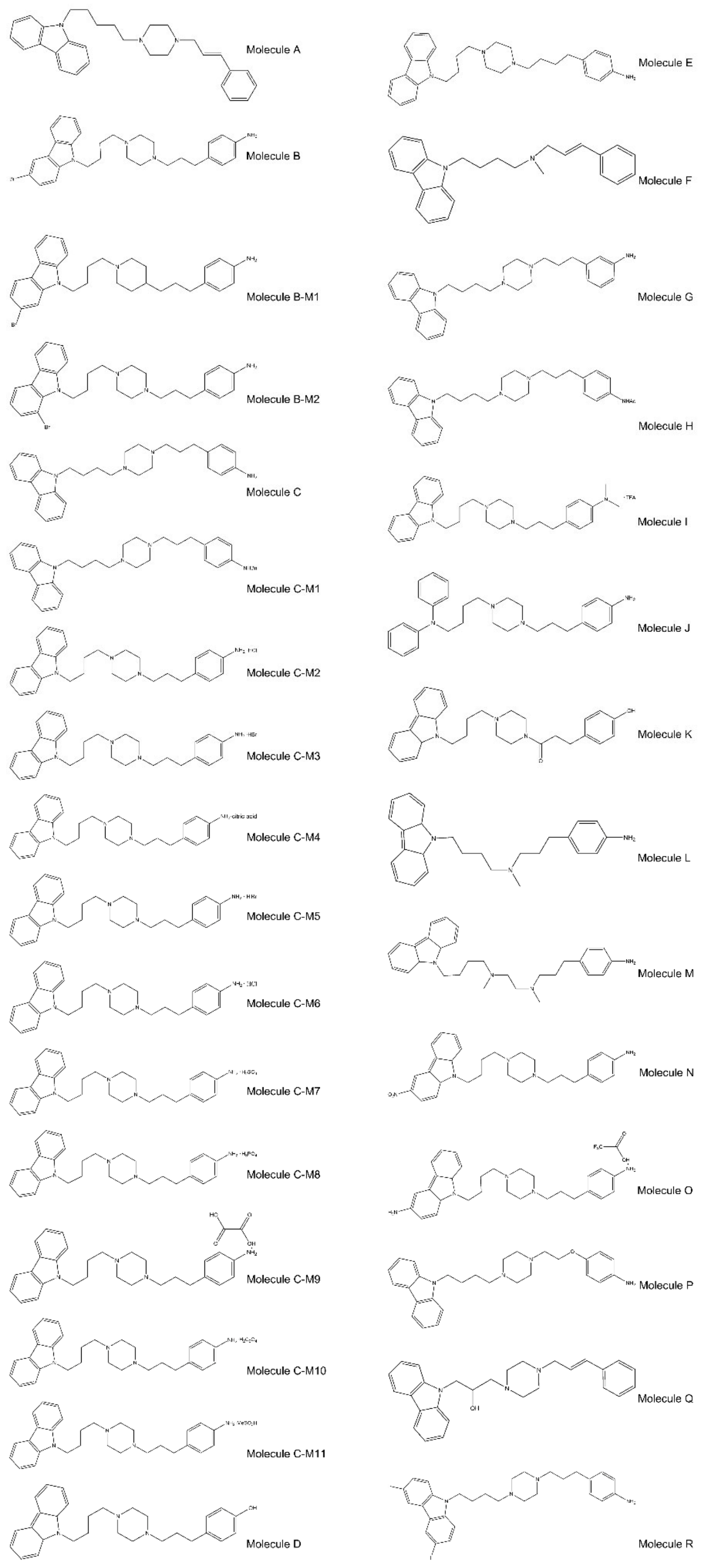

Figure S4. Chemical structures of 31 carbazole derivatives
