# Supplementary material for: Development of Carbazole Derivatives Compounds against Candida albicans: Candidates to Prevent Hyphal Formation via the Ras1-MAPK Pathway
Source: J Fungi (Basel). 2021 Aug 25;7(9):688. doi: 10.3390/jof7090688 (PMC8466151; doi:10.3390/jof7090688)
Supplement: Supplementary file 1 [file jof-07-00688-s001.zip › jof-1310716-supplementary/Supplementary table1.pdf]

Supplementary Table S1. Oligonucleotides used in this study.

Primer	Sequence (5` - 3`)	Purpose
UME6::HIS1 (F)	TTGTCATTCGTTTAAACAACAACTCACTTATTACTGTTTAATATAA AAACGAGGGATATCAATAACCTATATTCAGGAATTCATATTCTAT ATCTTCACTTTTAGGCTTTCTGACTTTCTTTTATGGCAAACA	ume6 deletion (HIS1 cassette) (forward)
UME6::HIS1 (R)	GCTATGTAAATTAAAACCTTGACTTGTTGGAATCTTCTACGGGAA AAGTTGCAAGAGTTGGTGTAAAATCAAATGGACCAAAATATTAC GAGAATATTAATACGCTGCATTTTACACAACATCTTTGTTAGATA	ume6 deletion (HIS1 cassette) (reverse)
Confirm UME6::HIS1 (F)	ATTCAGGAATTCATATTCTATATCTTCACT	ume6 deletion confirm (forward)
Confirm UME6::HIS1 (R)	AACCTTGACTTGTTGGAATCTT	ume6 deletion confirm (reverse)
NRG1::HIS1 (F)	AGCTTTCTTATTATTTACAACTGAGAATTAAAAATATCAAAAATA ATCCTATTATCAGTTATTATATACTAGGATTTGTTATTACAATTTG CTTTACAAGTTTAGGCTTTCTGACTTTCTTTTATGGCAAACA	nrg1 deletion (HIS1 cassette) (forward)
NRG1::HIS1 (R)	TGTTTGAAGAGCGCTTACTACACGGGGTTGTCTTAATACAAAGGC GGCAGGGTTGCAGTACTTCTGTAGTTTCTAACCTTTGTATTCCTTA GGCCCTGGATTGAACTCCCTTATGGTGCTCATGG	nrg1 deletion (HIS1 cassette) (reverse)
Confirm NRG1::HIS1 (F)	GGATTTGTTATTACAATTTGCTTTACAAG	nrg1 deletion confirm (forward)
Confirm NRG1::HIS1 (R)	TGTATTCCTTAGGCCCTGGA	nrg1 deletion confirm (reverse)
UME6-9myc-NAT1 (F)	AAGACAAAAGAAATTAACTGAAGTTTCATTGATTAGGAAAAGA AATCAAGCTAAAACAAAAATTTCTCGGAAAAAATCAAGTAATG ATATAACCAATGATtccggttctgctgctagtgg	UME6-9myc-NAT1 cassette (forward)
UME6-9myc-NAT1 (R)	AAATTATGAACAATTAATTAATCAATTAATAATAATAGTAATAG TAATAGTAATAAAAACAGTATAAAAATCAATCTTCCATTTTTTTT TCATTTTTTCTctagtgatctacgagaccgacacc	UME6-9myc-NAT1 cassette (reverse)
Confirm UME6-9myc-NAT1 (F)	CTGAAGTTTCATTGATTAGGAAAAGAAAT	UME6-9myc tagging confirm (forward)
Confirm UME6-9myc-NAT1 (R)	TGTGTGCAATATATTATATAAGAACAAAACAAC	UME6-9myc tagging confirm (reverse)
NRG1-9myc-NAT1 (F)	"CAAGACTCATACCAATGGGAAAAACAAACGCAACAGACAACA ACACAGGACTTTAGAAGCTTCACATGTTGGAACAAAATACAACA CCAAGAGCCTAGTAtccggttctgctgctagtgg	NRG1-9myc-NAT1 cassette (forward)
NRG1-9myc-NAT1 (R)	TATAATACTTCCTGTAGTAAATGTCGGAGTTTAAATTGCTGACAT TGCAAGAAAATAAAACCAATATAATATTTTTTATGTCACGAAAG AAATGGAACAActagtgatctacgagaccgacacc	NRG1-9myc-NAT1 cassette (reverse)
Confirm NRG1-9myc-NAT1 (F)	CAAACGCAACAGACAACAA	NRG1-9myc tagging confirm (forward)
Confirm NRG1-9myc-NAT1 (R)	TATAATACTTCCTGTAGTAAATGTCGG	NRG1-9myc tagging confirm (reverse)

UME6-F	GGTGGTGTCTCTTCTGATGTT	For quantitative PCR
UME6-R	CTACCACCACCATTGTTCAAATC	For quantitative PCR
NRG1-F	CAGTATGCTGCTCCTCCTTATC	For quantitative PCR
NRG1-R	GGTCTTTGCTTTGGGTGTTATT	
RAS1-F	GTGCTGTTAATGGTGGTGTAAG	For quantitative PCR
RAS1-R	GGAAGATTTGCTTGACCAGAAG	For quantitative PCR
ECE1-F	CCAAAATTGCCTGTGCTACTG	For quantitative PCR
ECE1-R	AACAGTAGGTGCTTGGTCAG	For quantitative PCR
ALS3-F	GTGATGCTGGATCTAACGGTATT	For quantitative PCR
ALS3-R	CGGTTAGGATCGAATGGTAAGG	For quantitative PCR
HWP1-F	GCCTGATGACAATCCTCCTATT	For quantitative PCR
HWP1-R	GAGTAGTAGCTGGAGTTGTTGG	For quantitative PCR
HGC1-F	ATATACACCAGGTCGCAAGC	For quantitative PCR
HGC1-R	AGAAACAGCACGAGAACCAG	For quantitative PCR
CYR1-F	CCTCCATTGGCTCAACCTATAA	For quantitative PCR
CYR1-R	GGCTGTTTCATCGTCTTCTTTC	For quantitative PCR
CST20-F	CCCTGATGACGCTGCATTTA	For quantitative PCR
CST20-R	GGTGTTGCTGGTGGAGTATTT	For quantitative PCR
TPK1-F	AACTTCTGGTGTTGGTGATACT	For quantitative PCR
TPK1-R	TGGGTCTTCAACTCCACTTATAC	For quantitative PCR
STE11-F	TGGCACCTGAAGTGGTTAAG	For quantitative PCR
STE11-R	AAATCTGGGAATGGATGTCTACC	For quantitative PCR
HST7-F	CAGTATGGTCGGAGGATGATAAC	For quantitative PCR
HST7-R	TCTGCTTCTGCTTGGACTATTT	For quantitative PCR
CEK1-F	GCAGCTACTACTTCTTCTTCCC	For quantitative PCR

CEK1-R	TTCCATATGCTCCTTCTCCAAC	For quantitative PCR
MKC1-F	GGCTAAACCATCAGGAGAAGAG	For quantitative PCR
MKC1-R	CTGGTGGTCGTTACAGTAGTTG	For quantitative PCR
ACT1-F	TGAAGCCCAATCCAAAAGAGG	For quantitative PCR
ACT1-R	TTTCCATATCGTCCCAGTTGG	For quantitative PCR
