# Supplementary figures and images for: Development of Carbazole Derivatives Compounds against Candida albicans: Candidates to Prevent Hyphal Formation via the Ras1-MAPK Pathway
Source: J Fungi (Basel). 2021 Aug 25;7(9):688. doi: 10.3390/jof7090688 (PMC8466151; doi:10.3390/jof7090688)

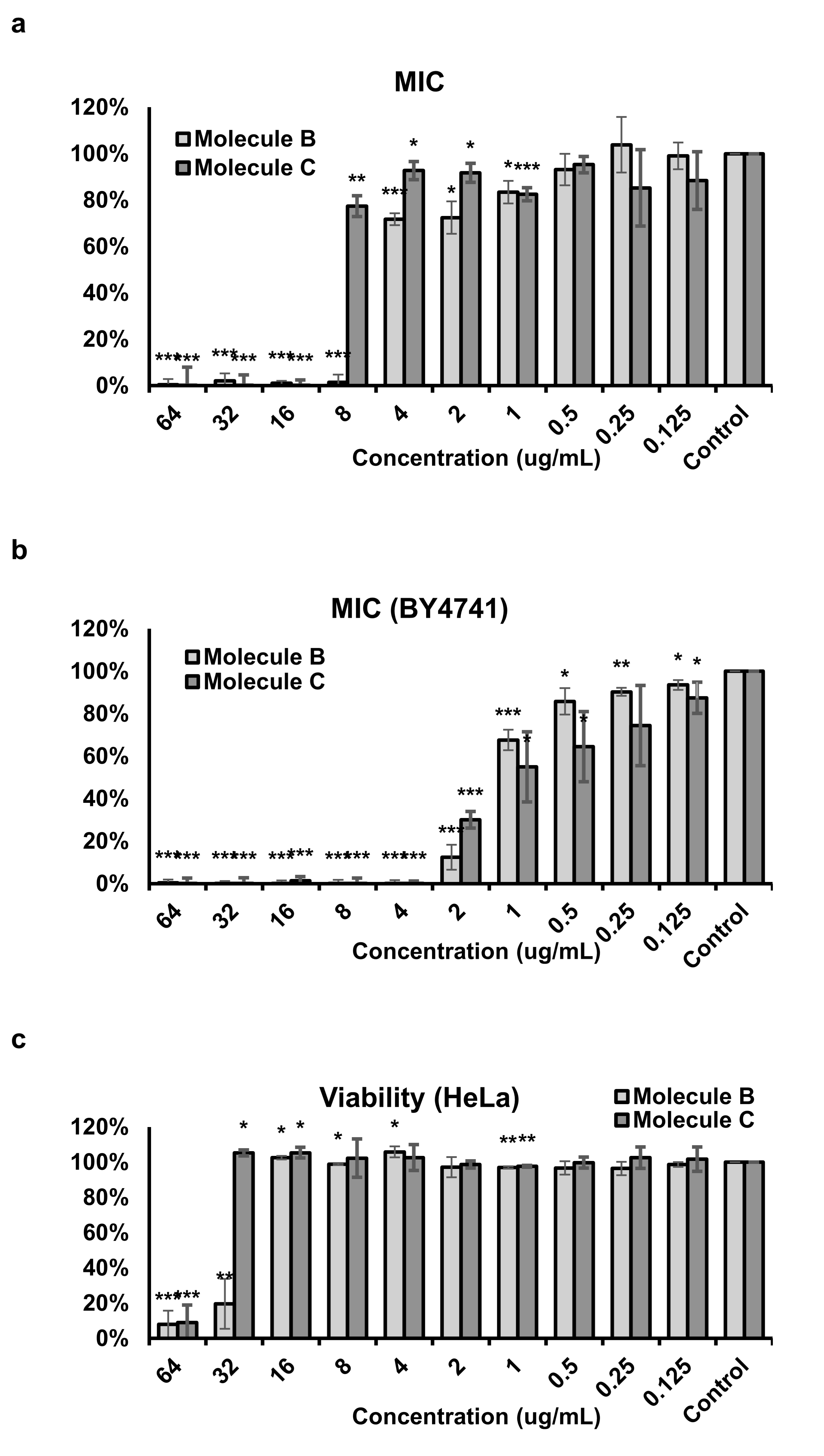

Supplement: Supplementary file 1 [file jof-07-00688-s001.zip › jof-1310716-supplementary/Figure S1.tif]

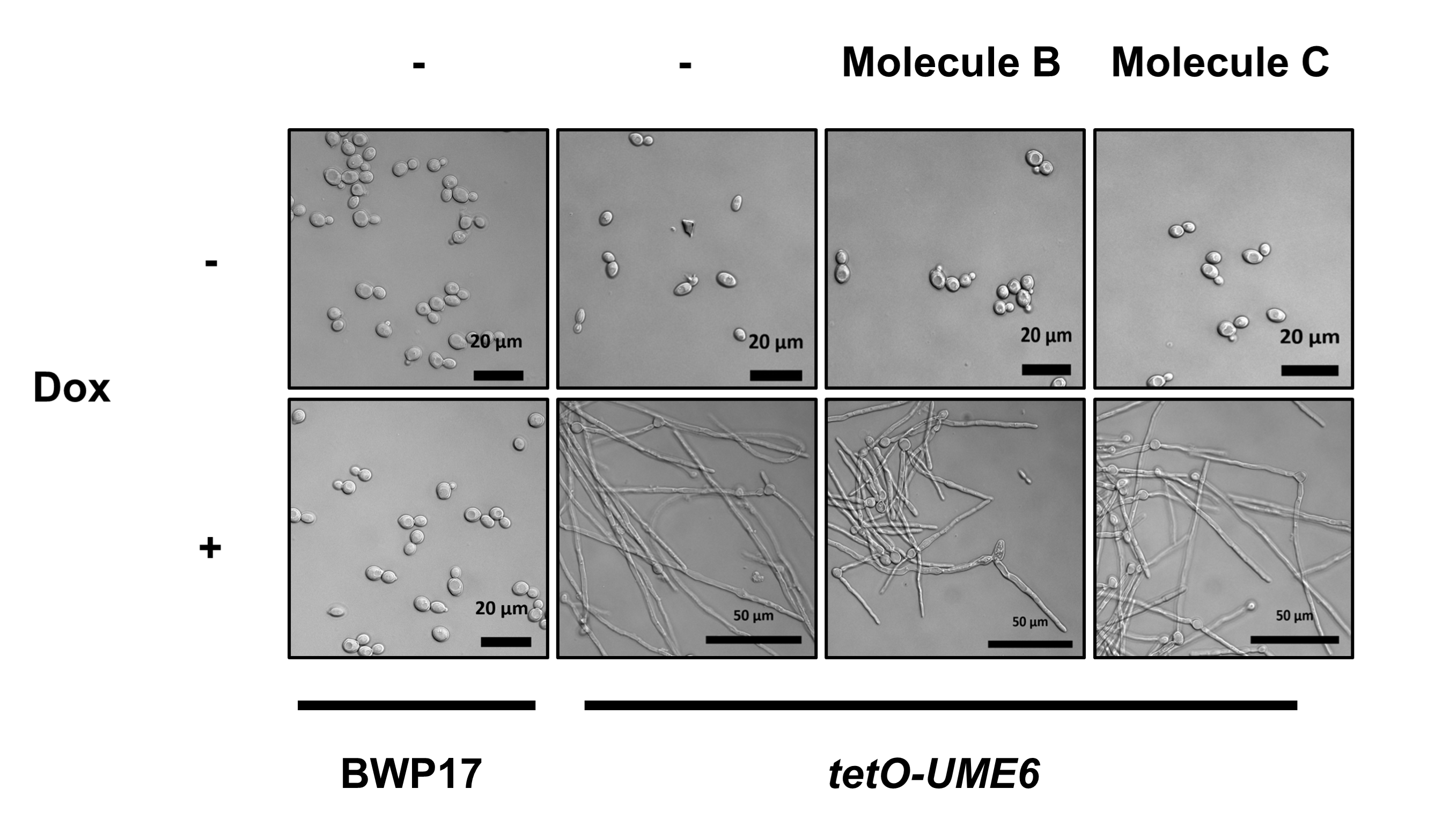

Supplement: Supplementary file 1 [file jof-07-00688-s001.zip › jof-1310716-supplementary/Figure S2.tif]

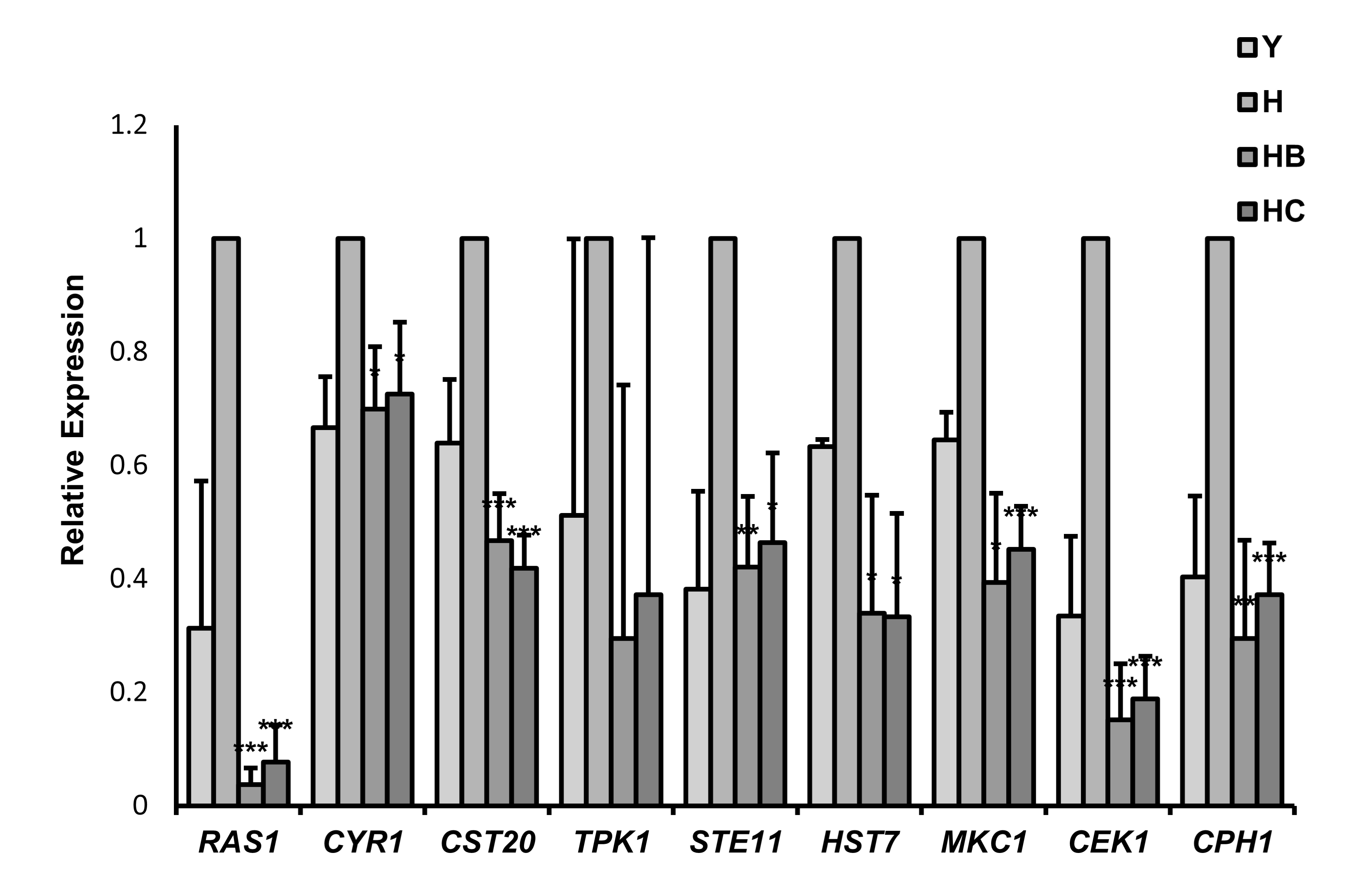

Supplement: Supplementary file 1 [file jof-07-00688-s001.zip › jof-1310716-supplementary/Figure S3.tif]
